# *N*-{(1*Z*)-1-[(6-Chloro­pyridin-3-ylmeth­yl)(eth­yl)amino]-3-(3-chloro­phen­yl)-2-nitro-5-oxohex-1-en­yl}-*N*-methyl­acetamide

**DOI:** 10.1107/S1600536812043358

**Published:** 2012-10-27

**Authors:** Chuan-Wen Sun, Ying Wu, Jing Wang

**Affiliations:** a Department of Chemistry, College of Life and Environmental Science, Shanghai Normal University, Shanghai, 200234, People’s Republic of China

## Abstract

In the title compound, C_23_H_26_Cl_2_N_4_O_4_,the dihedral angle between the mean planes of the pyridine and 3-chloro­phenyl rings is 22.63 (2)°. The nitro group is in a *Z* conformation.

## Related literature

For general background to neonicotinoid compounds and their application as insecticides, see: Tomizawa & Casida, (2000 ▶); Minamida *et al.* (1993 ▶); Kashiwada *et al.* (1996 ▶). For the synthesis, see: Zhang *et al.* (2010 ▶).
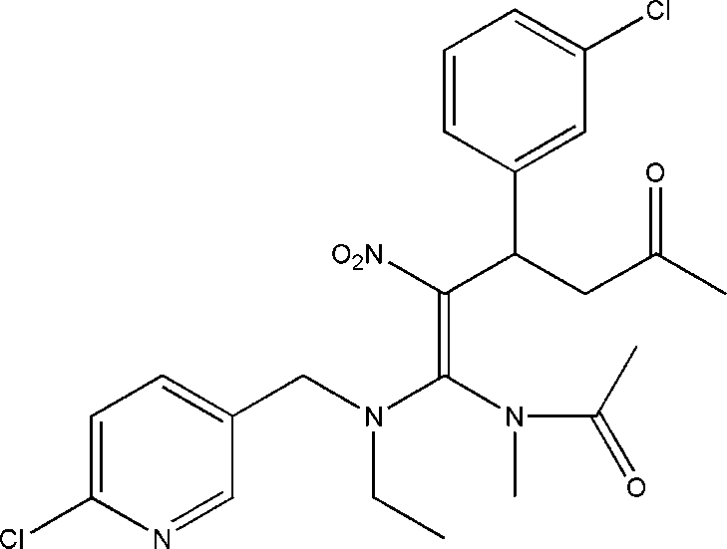


## Experimental

### 

#### Crystal data

C_23_H_26_Cl_2_N_4_O_4_
*M* *_r_* = 493.38
Triclinic, 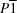

*a* = 7.7948 (13) Å
*b* = 12.649 (2) Å
*c* = 13.021 (2) Å
α = 91.364 (3)°
β = 98.765 (2)°
γ = 107.878 (3)°
*V* = 1204.1 (3) Å^3^
*Z* = 2
Mo *K*α radiation
μ = 0.31 mm^−1^
*T* = 298 K
0.16 × 0.12 × 0.10 mm

#### Data collection

Bruker SMART APEX CCD area-detector diffractometer
Absorption correction: multi-scan (*SADABS*; Sheldrick, 1996 ▶) *T*_min_ = 0.943, *T*_max_ = 0.970
7096 measured reflections
4200 independent reflections
3870 reflections with *I* > 2σ(*I*)
*R*_int_ = 0.020

#### Refinement

*R*[*F* ^2^ > 2σ(*F* ^2^)] = 0.064
*wR*(*F* ^2^) = 0.148
*S* = 1.17
4200 reflections
302 parameters
H-atom parameters constrained
Δρ_max_ = 0.47 e Å^−3^
Δρ_min_ = −0.38 e Å^−3^

### 

Data collection: *SMART* (Bruker, 2002 ▶); cell refinement: *SAINT* (Bruker, 2002 ▶); data reduction: *SAINT*; program(s) used to solve structure: *SHELXS97* (Sheldrick, 2008 ▶); program(s) used to refine structure: *SHELXL97* (Sheldrick, 2008 ▶); molecular graphics: *SHELXTL* (Sheldrick, 2008 ▶); software used to prepare material for publication: *SHELXTL*.

## Supplementary Material

Crystal structure: contains datablock(s) I, global. DOI: 10.1107/S1600536812043358/jj2153sup1.cif

Structure factors: contains datablock(s) I. DOI: 10.1107/S1600536812043358/jj2153Isup2.hkl

Supplementary material file. DOI: 10.1107/S1600536812043358/jj2153Isup3.cml

Additional supplementary materials:  crystallographic information; 3D view; checkCIF report

## Figures and Tables

**Fig. 1.**
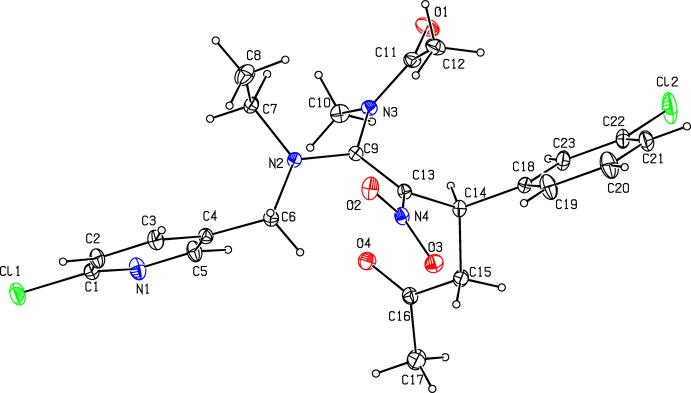
The molecular structure of (I), showing the atom-numbering scheme. Displacement ellipsoids are drawn at the 50% probability level.

## supplementary crystallographic information

### Comment

Neonicotinoid insecticides have gained worldwide attention for being the fastest growing class of insecticides in modern crop protection, with wide spread use against sucking and chewing pests. Since imidacloprid (IMI) was first introduced to the market in 1991, many new neonicotinoid insecticides (NNSs) are now being sold. Nitenpyram, a chloronicotinyl derivative marketed in 1995, was characterized with a much lower toxicity against the mammals than imidacloprid (Tomizawa & Casida, 2000; Minamida *et al.*, 1993; Kashiwada *et al.*, 1996). In this paper, the title compound, (I), a new derivative, has been synthesized and characterized by X-ray diffraction.

In the title compound, C_23_H_26_Cl_2_N_4_O_4_, there is one molecule in the asymmetric unit (Fig. 1). The dihedral angle between the mean planes of the pyridine and 3-chlorophenyl rings is 22.63 (2)°. As compared with the *(E)* configuration of the nitro group in the crystal structure of nitenpyram, the nitro group in the title compound is in the *(Z)* configuration as anticipated.

### Experimental

The title compound was prepared by the literature method (Zhang *et al.*, 2010). It was obtained using volatilization of petroleum ether and ethyl acetate solution at room temperature, giving yellow crystals (yield 78.6%). Anal. calcd. for C_23_H_26_Cl_2_N_4_O_4_ C 55.99, H 5.31, N 11.36%. found, C 55.97, H 5.32, N 11.38%.

### Refinement

In (I), H atoms bonded to C and N atoms were located at their ideal positions and subsequently treated as riding modes with C–H distances of 0.93Å (aromatic), 0.97Å (methylene), 0.98Å (methine) and 0.96Å (methyl) with *U*_iso_(H) = 1.2*U*_eq_(aromatic, methylene methine C) or 1.5*U*_eq_(methyl C).

### Crystal data

**Table:**

C_23_H_26_Cl_2_N_4_O_4_	*Z* = 2
*M**_r_* = 493.38	*F*(000) = 516
Triclinic, *P*1	*D*_x_ = 1.361 Mg m^−^^3^
Hall symbol: -P 1	Mo *K*α radiation, λ = 0.71073 Å
*a* = 7.7948 (13) Å	Cell parameters from 4525 reflections
*b* = 12.649 (2) Å	θ = 2.2–28.3°
*c* = 13.021 (2) Å	µ = 0.31 mm^−^^1^
α = 91.364 (3)°	*T* = 298 K
β = 98.765 (2)°	Block, yellow
γ = 107.878 (3)°	0.16 × 0.12 × 0.10 mm
*V* = 1204.1 (3) Å^3^	

### Data collection

**Table:**

Bruker SMART APEX CCD area-detector diffractometer	4200 independent reflections
Radiation source: fine-focus sealed tube	3870 reflections with *I* > 2σ(*I*)
Graphite monochromator	*R*_int_ = 0.020
phi and ω scans	θ_max_ = 25.0°, θ_min_ = 2.2°
Absorption correction: multi-scan (*SADABS*; Sheldrick, 1996)	*h* = −7→9
*T*_min_ = 0.943, *T*_max_ = 0.970	*k* = −15→13
7096 measured reflections	*l* = −15→15

### Refinement

**Table:**

Refinement on *F*^2^	Primary atom site location: structure-invariant direct methods
Least-squares matrix: full	Secondary atom site location: difference Fourier map
*R*[*F*^2^ > 2σ(*F*^2^)] = 0.064	Hydrogen site location: inferred from neighbouring sites
*wR*(*F*^2^) = 0.148	H-atom parameters constrained
*S* = 1.17	*w* = 1/[σ^2^(*F*_o_^2^) + (0.0453*P*)^2^ + 0.9109*P*] where *P* = (*F*_o_^2^ + 2*F*_c_^2^)/3
4200 reflections	(Δ/σ)_max_ = 0.011
302 parameters	Δρ_max_ = 0.47 e Å^−^^3^
0 restraints	Δρ_min_ = −0.38 e Å^−^^3^

### Special details

**Table:**

Experimental. ^1^H NMR (CDCl_3_, 400 Hz): 8.47 (d, *J* = 1.9 Hz,1*H*, Py—H), 7.83 (dd, *J* = 8.2, 2.4 Hz, 1*H*, Py—H), 7.42 (d, *J* = 8.2 Hz, 1*H*, Py—H), 7.12 (d, *J* = 10.2 Hz, 1*H*, Ph—H), 7.01–6.91 (m, 3*H*, Ph—H), 4.27 (d, *J* = 14.7 Hz, 1*H*), 4.12 (d, *J* = 7.7 Hz, 1*H*), 4.00 (dd, *J* = 10.8, 3.8 Hz, 1*H*), 3.78 (d, *J* = 14.8 Hz, 1*H*), 3.31 (s, 3*H*, NCH_3_), 3.09 (dd, *J* = 14.4, 7.2 Hz, 1*H*, NCH_2_), 2.89 (d, *J* = 3.9Hz, 1*H*, NCH_2_), 2.26 (s, 1*H*), 2.13 (s, 3*H*), 1.67 (s, 3*H*), 1.17 (t, J = 7.2 Hz, 3*H*, NCH_2_CH_3_). IR(KBr, cm^-1^) 2945 (CH_3_), 1710 (C=O), 1658 (C=C), 1378, 1390(NO_2_), 1660, 1621, 1538(benzene).
Geometry. All esds (except the esd in the dihedral angle between two l.s. planes) are estimated using the full covariance matrix. The cell esds are taken into account individually in the estimation of esds in distances, angles and torsion angles; correlations between esds in cell parameters are only used when they are defined by crystal symmetry. An approximate (isotropic) treatment of cell esds is used for estimating esds involving l.s. planes.
Refinement. Refinement of *F*^2^ against ALL reflections. The weighted *R*-factor *wR* and goodness of fit *S* are based on *F*^2^, conventional *R*-factors *R* are based on *F*, with *F* set to zero for negative *F*^2^. The threshold expression of *F*^2^ > σ(*F*^2^) is used only for calculating *R*-factors(gt) *etc*. and is not relevant to the choice of reflections for refinement. *R*-factors based on *F*^2^ are statistically about twice as large as those based on *F*, and *R*- factors based on ALL data will be even larger.

### Fractional atomic coordinates and isotropic or equivalent isotropic displacement parameters (Å^2^)

**Table:**

	*x*	*y*	*z*	*U*_iso_*/*U*_eq_	
C1	0.9628 (4)	0.8187 (2)	0.8649 (3)	0.0521 (7)	
C2	0.9304 (4)	0.8215 (3)	0.7596 (2)	0.0559 (8)	
H2	1.0030	0.8779	0.7259	0.067*	
C3	0.7857 (4)	0.7376 (3)	0.7052 (2)	0.0540 (8)	
H3	0.7581	0.7367	0.6331	0.065*	
C4	0.6813 (4)	0.6546 (2)	0.7573 (2)	0.0427 (6)	
C5	0.7293 (4)	0.6599 (3)	0.8637 (2)	0.0596 (8)	
H5	0.6613	0.6036	0.8995	0.071*	
C6	0.5256 (4)	0.5591 (2)	0.6992 (2)	0.0451 (6)	
H6A	0.5205	0.5665	0.6249	0.054*	
H6B	0.5492	0.4896	0.7142	0.054*	
C7	0.3021 (4)	0.6592 (2)	0.7287 (2)	0.0480 (7)	
H7A	0.4057	0.7180	0.7664	0.058*	
H7B	0.1998	0.6501	0.7655	0.058*	
C8	0.2535 (6)	0.6925 (3)	0.6212 (3)	0.0785 (11)	
H8A	0.3540	0.7011	0.5843	0.118*	
H8B	0.2277	0.7618	0.6258	0.118*	
H8C	0.1475	0.6360	0.5846	0.118*	
C9	0.2212 (3)	0.4559 (2)	0.73211 (18)	0.0348 (5)	
C10	0.1460 (5)	0.4994 (3)	0.9036 (2)	0.0550 (8)	
H10A	0.0962	0.5579	0.9175	0.083*	
H10B	0.2771	0.5277	0.9188	0.083*	
H10C	0.1029	0.4395	0.9465	0.083*	
C11	−0.0970 (4)	0.4176 (3)	0.7564 (2)	0.0519 (7)	
C12	−0.1607 (4)	0.3915 (3)	0.6418 (3)	0.0650 (9)	
H12A	−0.2290	0.3138	0.6283	0.098*	
H12B	−0.0569	0.4085	0.6066	0.098*	
H12C	−0.2373	0.4353	0.6169	0.098*	
C13	0.2214 (3)	0.3532 (2)	0.69263 (19)	0.0368 (6)	
C14	0.1557 (4)	0.2493 (2)	0.7497 (2)	0.0421 (6)	
H14	0.1153	0.2752	0.8105	0.051*	
C15	0.3084 (4)	0.2029 (2)	0.7966 (2)	0.0542 (7)	
H15A	0.2532	0.1285	0.8171	0.065*	
H15B	0.3801	0.1969	0.7433	0.065*	
C16	0.4344 (4)	0.2730 (2)	0.8897 (2)	0.0500 (7)	
C17	0.5633 (6)	0.2214 (3)	0.9493 (3)	0.0795 (11)	
H17A	0.6664	0.2789	0.9873	0.119*	
H17B	0.6046	0.1792	0.9019	0.119*	
H17C	0.5019	0.1731	0.9972	0.119*	
C18	−0.0129 (4)	0.1572 (2)	0.6946 (2)	0.0454 (6)	
C19	−0.0443 (5)	0.1205 (3)	0.5905 (3)	0.0766 (11)	
H19	0.0405	0.1535	0.5483	0.092*	
C20	−0.2016 (6)	0.0346 (4)	0.5483 (3)	0.0891 (13)	
H20	−0.2201	0.0106	0.4782	0.107*	
C21	−0.3293 (5)	−0.0151 (3)	0.6078 (3)	0.0746 (11)	
H21	−0.4345	−0.0724	0.5793	0.090*	
C22	−0.2985 (5)	0.0214 (3)	0.7100 (3)	0.0661 (9)	
C23	−0.1431 (4)	0.1059 (2)	0.7539 (3)	0.0581 (8)	
H23	−0.1258	0.1286	0.8242	0.070*	
Cl1	1.14377 (13)	0.92531 (8)	0.93748 (8)	0.0793 (3)	
Cl2	−0.45945 (18)	−0.04123 (10)	0.78826 (12)	0.1249 (6)	
N1	0.8686 (4)	0.7416 (2)	0.9194 (2)	0.0676 (8)	
N2	0.3480 (3)	0.55437 (17)	0.72679 (16)	0.0377 (5)	
N3	0.0879 (3)	0.45855 (18)	0.79346 (17)	0.0416 (5)	
N4	0.2894 (3)	0.34678 (19)	0.59894 (18)	0.0459 (6)	
O1	−0.2039 (3)	0.4098 (2)	0.8168 (2)	0.0819 (8)	
O2	0.2890 (3)	0.41880 (18)	0.53559 (15)	0.0616 (6)	
O3	0.3403 (3)	0.26542 (19)	0.57805 (18)	0.0650 (6)	
O4	0.4321 (3)	0.36466 (18)	0.91429 (17)	0.0595 (6)	

### Atomic displacement parameters (Å^2^)

**Table:**

	*U*^11^	*U*^22^	*U*^33^	*U*^12^	*U*^13^	*U*^23^
C1	0.0429 (16)	0.0456 (16)	0.0630 (19)	0.0094 (13)	0.0035 (14)	0.0074 (14)
C2	0.0512 (17)	0.0492 (17)	0.0596 (19)	0.0001 (14)	0.0176 (15)	0.0138 (14)
C3	0.0560 (18)	0.0566 (18)	0.0424 (16)	0.0040 (14)	0.0158 (14)	0.0045 (13)
C4	0.0409 (14)	0.0416 (14)	0.0454 (15)	0.0103 (12)	0.0126 (12)	0.0029 (12)
C5	0.0566 (19)	0.0544 (18)	0.0537 (19)	−0.0035 (15)	0.0088 (15)	0.0142 (14)
C6	0.0449 (15)	0.0438 (15)	0.0470 (16)	0.0104 (12)	0.0166 (12)	−0.0009 (12)
C7	0.0543 (17)	0.0365 (14)	0.0537 (17)	0.0151 (13)	0.0096 (13)	0.0014 (12)
C8	0.104 (3)	0.062 (2)	0.072 (2)	0.034 (2)	0.004 (2)	0.0170 (18)
C9	0.0377 (13)	0.0398 (13)	0.0273 (12)	0.0119 (11)	0.0069 (10)	0.0048 (10)
C10	0.0659 (19)	0.0668 (19)	0.0377 (15)	0.0243 (16)	0.0184 (14)	−0.0012 (13)
C11	0.0449 (16)	0.0550 (17)	0.0609 (19)	0.0194 (14)	0.0162 (14)	0.0080 (14)
C12	0.0472 (18)	0.080 (2)	0.066 (2)	0.0234 (17)	−0.0005 (15)	−0.0007 (18)
C13	0.0363 (13)	0.0391 (14)	0.0337 (13)	0.0075 (11)	0.0109 (10)	0.0009 (10)
C14	0.0458 (15)	0.0363 (14)	0.0398 (14)	0.0068 (11)	0.0067 (12)	0.0007 (11)
C15	0.0574 (18)	0.0411 (15)	0.0588 (18)	0.0132 (13)	−0.0013 (15)	0.0030 (13)
C16	0.0471 (16)	0.0479 (17)	0.0512 (17)	0.0095 (13)	0.0079 (13)	0.0063 (13)
C17	0.084 (3)	0.064 (2)	0.079 (3)	0.0224 (19)	−0.022 (2)	0.0009 (18)
C18	0.0453 (15)	0.0348 (14)	0.0536 (17)	0.0101 (12)	0.0059 (13)	0.0043 (12)
C19	0.072 (2)	0.080 (2)	0.052 (2)	−0.0111 (19)	0.0038 (17)	0.0028 (17)
C20	0.086 (3)	0.089 (3)	0.057 (2)	−0.010 (2)	−0.013 (2)	−0.005 (2)
C21	0.055 (2)	0.053 (2)	0.094 (3)	−0.0023 (16)	−0.0104 (19)	0.0010 (19)
C22	0.0543 (19)	0.0384 (16)	0.098 (3)	0.0009 (14)	0.0198 (18)	−0.0028 (17)
C23	0.0622 (19)	0.0373 (15)	0.069 (2)	0.0038 (14)	0.0196 (16)	−0.0040 (14)
Cl1	0.0610 (5)	0.0625 (5)	0.0888 (7)	−0.0048 (4)	−0.0140 (5)	0.0050 (5)
Cl2	0.1075 (9)	0.0734 (7)	0.1642 (13)	−0.0375 (6)	0.0766 (9)	−0.0304 (7)
N1	0.0650 (18)	0.0645 (17)	0.0553 (16)	−0.0018 (14)	0.0001 (13)	0.0126 (13)
N2	0.0418 (12)	0.0355 (11)	0.0369 (11)	0.0115 (9)	0.0115 (9)	0.0027 (9)
N3	0.0413 (12)	0.0477 (13)	0.0389 (12)	0.0151 (10)	0.0144 (10)	0.0016 (10)
N4	0.0456 (13)	0.0450 (13)	0.0420 (13)	0.0053 (10)	0.0122 (10)	−0.0092 (11)
O1	0.0523 (14)	0.118 (2)	0.0845 (18)	0.0287 (14)	0.0336 (13)	0.0132 (16)
O2	0.0865 (16)	0.0549 (13)	0.0370 (11)	0.0071 (11)	0.0221 (11)	0.0050 (10)
O3	0.0679 (14)	0.0622 (14)	0.0711 (15)	0.0237 (11)	0.0270 (12)	−0.0149 (11)
O4	0.0589 (13)	0.0600 (14)	0.0574 (13)	0.0196 (11)	0.0041 (10)	−0.0119 (10)

### Geometric parameters (Å, º)

**Table:**

C1—N1	1.321 (4)	C11—C12	1.494 (4)
C1—C2	1.359 (4)	C12—H12A	0.9600
C1—Cl1	1.751 (3)	C12—H12B	0.9600
C2—C3	1.373 (4)	C12—H12C	0.9600
C2—H2	0.9300	C13—N4	1.412 (3)
C3—C4	1.379 (4)	C13—C14	1.513 (4)
C3—H3	0.9300	C14—C18	1.527 (4)
C4—C5	1.374 (4)	C14—C15	1.536 (4)
C4—C6	1.510 (4)	C14—H14	0.9800
C5—N1	1.344 (4)	C15—C16	1.508 (4)
C5—H5	0.9300	C15—H15A	0.9700
C6—N2	1.466 (3)	C15—H15B	0.9700
C6—H6A	0.9700	C16—O4	1.202 (3)
C6—H6B	0.9700	C16—C17	1.492 (5)
C7—N2	1.476 (3)	C17—H17A	0.9600
C7—C8	1.498 (4)	C17—H17B	0.9600
C7—H7A	0.9700	C17—H17C	0.9600
C7—H7B	0.9700	C18—C23	1.380 (4)
C8—H8A	0.9600	C18—C19	1.383 (4)
C8—H8B	0.9600	C19—C20	1.391 (5)
C8—H8C	0.9600	C19—H19	0.9300
C9—N2	1.343 (3)	C20—C21	1.364 (6)
C9—C13	1.387 (3)	C20—H20	0.9300
C9—N3	1.412 (3)	C21—C22	1.359 (5)
C10—N3	1.467 (3)	C21—H21	0.9300
C10—H10A	0.9600	C22—C23	1.378 (4)
C10—H10B	0.9600	C22—Cl2	1.749 (4)
C10—H10C	0.9600	C23—H23	0.9300
C11—O1	1.215 (4)	N4—O2	1.244 (3)
C11—N3	1.377 (4)	N4—O3	1.249 (3)
			
N1—C1—C2	125.7 (3)	C9—C13—C14	121.2 (2)
N1—C1—Cl1	115.7 (2)	N4—C13—C14	119.8 (2)
C2—C1—Cl1	118.6 (2)	C13—C14—C18	117.0 (2)
C1—C2—C3	117.1 (3)	C13—C14—C15	114.0 (2)
C1—C2—H2	121.4	C18—C14—C15	111.8 (2)
C3—C2—H2	121.4	C13—C14—H14	104.1
C2—C3—C4	120.2 (3)	C18—C14—H14	104.1
C2—C3—H3	119.9	C15—C14—H14	104.1
C4—C3—H3	119.9	C16—C15—C14	113.8 (2)
C5—C4—C3	117.3 (3)	C16—C15—H15A	108.8
C5—C4—C6	121.4 (2)	C14—C15—H15A	108.8
C3—C4—C6	121.3 (3)	C16—C15—H15B	108.8
N1—C5—C4	124.1 (3)	C14—C15—H15B	108.8
N1—C5—H5	118.0	H15A—C15—H15B	107.7
C4—C5—H5	118.0	O4—C16—C17	122.0 (3)
N2—C6—C4	112.9 (2)	O4—C16—C15	122.3 (3)
N2—C6—H6A	109.0	C17—C16—C15	115.7 (3)
C4—C6—H6A	109.0	C16—C17—H17A	109.5
N2—C6—H6B	109.0	C16—C17—H17B	109.5
C4—C6—H6B	109.0	H17A—C17—H17B	109.5
H6A—C6—H6B	107.8	C16—C17—H17C	109.5
N2—C7—C8	112.1 (2)	H17A—C17—H17C	109.5
N2—C7—H7A	109.2	H17B—C17—H17C	109.5
C8—C7—H7A	109.2	C23—C18—C19	117.6 (3)
N2—C7—H7B	109.2	C23—C18—C14	117.3 (3)
C8—C7—H7B	109.2	C19—C18—C14	125.2 (3)
H7A—C7—H7B	107.9	C18—C19—C20	120.6 (3)
C7—C8—H8A	109.5	C18—C19—H19	119.7
C7—C8—H8B	109.5	C20—C19—H19	119.7
H8A—C8—H8B	109.5	C21—C20—C19	121.2 (4)
C7—C8—H8C	109.5	C21—C20—H20	119.4
H8A—C8—H8C	109.5	C19—C20—H20	119.4
H8B—C8—H8C	109.5	C22—C21—C20	118.1 (3)
N2—C9—C13	126.1 (2)	C22—C21—H21	120.9
N2—C9—N3	115.3 (2)	C20—C21—H21	120.9
C13—C9—N3	118.3 (2)	C21—C22—C23	121.8 (3)
N3—C10—H10A	109.5	C21—C22—Cl2	119.1 (3)
N3—C10—H10B	109.5	C23—C22—Cl2	119.1 (3)
H10A—C10—H10B	109.5	C22—C23—C18	120.7 (3)
N3—C10—H10C	109.5	C22—C23—H23	119.6
H10A—C10—H10C	109.5	C18—C23—H23	119.6
H10B—C10—H10C	109.5	C1—N1—C5	115.6 (3)
O1—C11—N3	119.2 (3)	C9—N2—C6	120.5 (2)
O1—C11—C12	121.7 (3)	C9—N2—C7	121.1 (2)
N3—C11—C12	119.0 (3)	C6—N2—C7	117.4 (2)
C11—C12—H12A	109.5	C11—N3—C9	122.8 (2)
C11—C12—H12B	109.5	C11—N3—C10	117.8 (2)
H12A—C12—H12B	109.5	C9—N3—C10	119.3 (2)
C11—C12—H12C	109.5	O2—N4—O3	120.6 (2)
H12A—C12—H12C	109.5	O2—N4—C13	119.8 (2)
H12B—C12—H12C	109.5	O3—N4—C13	119.5 (2)
C9—C13—N4	118.9 (2)		
			
N1—C1—C2—C3	0.7 (5)	C20—C21—C22—C23	−0.3 (6)
Cl1—C1—C2—C3	−179.0 (2)	C20—C21—C22—Cl2	−179.7 (3)
C1—C2—C3—C4	−0.6 (5)	C21—C22—C23—C18	0.5 (5)
C2—C3—C4—C5	−0.4 (5)	Cl2—C22—C23—C18	179.9 (3)
C2—C3—C4—C6	−177.9 (3)	C19—C18—C23—C22	−0.2 (5)
C3—C4—C5—N1	1.3 (5)	C14—C18—C23—C22	−179.5 (3)
C6—C4—C5—N1	178.7 (3)	C2—C1—N1—C5	0.1 (5)
C5—C4—C6—N2	64.1 (4)	Cl1—C1—N1—C5	179.8 (3)
C3—C4—C6—N2	−118.5 (3)	C4—C5—N1—C1	−1.1 (5)
N2—C9—C13—N4	−35.2 (4)	C13—C9—N2—C6	−14.8 (4)
N3—C9—C13—N4	152.1 (2)	N3—C9—N2—C6	158.1 (2)
N2—C9—C13—C14	143.0 (3)	C13—C9—N2—C7	153.5 (3)
N3—C9—C13—C14	−29.7 (4)	N3—C9—N2—C7	−33.6 (3)
C9—C13—C14—C18	116.5 (3)	C4—C6—N2—C9	−143.8 (2)
N4—C13—C14—C18	−65.4 (3)	C4—C6—N2—C7	47.4 (3)
C9—C13—C14—C15	−110.5 (3)	C8—C7—N2—C9	−94.4 (3)
N4—C13—C14—C15	67.7 (3)	C8—C7—N2—C6	74.3 (3)
C13—C14—C15—C16	71.6 (3)	O1—C11—N3—C9	170.8 (3)
C18—C14—C15—C16	−152.9 (3)	C12—C11—N3—C9	−13.3 (4)
C14—C15—C16—O4	−10.8 (4)	O1—C11—N3—C10	−4.9 (4)
C14—C15—C16—C17	169.4 (3)	C12—C11—N3—C10	170.9 (3)
C13—C14—C18—C23	−137.1 (3)	N2—C9—N3—C11	126.2 (3)
C15—C14—C18—C23	88.9 (3)	C13—C9—N3—C11	−60.3 (3)
C13—C14—C18—C19	43.7 (4)	N2—C9—N3—C10	−58.1 (3)
C15—C14—C18—C19	−90.4 (4)	C13—C9—N3—C10	115.4 (3)
C23—C18—C19—C20	−0.3 (6)	C9—C13—N4—O2	−24.5 (4)
C14—C18—C19—C20	179.0 (3)	C14—C13—N4—O2	157.2 (2)
C18—C19—C20—C21	0.4 (7)	C9—C13—N4—O3	159.3 (2)
C19—C20—C21—C22	−0.2 (7)	C14—C13—N4—O3	−18.9 (4)
